# USP18 negatively regulates NF-κB signaling by targeting TAK1 and NEMO for deubiquitination through distinct mechanisms

**DOI:** 10.1038/srep12738

**Published:** 2015-08-04

**Authors:** Zhifen Yang, Huifang Xian, Jiajia Hu, Shuo Tian, Yunfei Qin, Rong-Fu Wang, Jun Cui

**Affiliations:** 1 Key Laboratory of Gene Engineering of the Ministry of Education, State Key Laboratory of Biocontrol, School of Life Sciences; 2 Zhongshan School of Medicine; 3 Collaborative Innovation Center of Cancer Medicine, Sun Yat-sen University, Guangzhou, P. R. China; 4 Houston Methodist Research Institute, Houston, Texas 77030, USA

## Abstract

Nuclear factor κB (NF-κB) is a key transcription factor in inflammatory immune responses and cell survival. Multiple types of ubiquitination play critical roles in the activation of NF-κB signaling, yet the molecular mechanisms responsible for their reversible deubiquitination are still poorly understood. In this study, we identified a member of the deubiquitinases family, ubiquitin-specific protease 18 (USP18), as a novel negative regulator in Toll-like receptor (TLR)-mediated NF-κB activation in human macrophages. USP18 is an interferon inducible gene, which is also upregulated by various TLR ligands in human monocytes and macrophages. Knockdown of USP18 enhanced the phosphorylation of IKK, the degradation of IκB, and augmented the expression of pro-inflammatory cytokines. Furthermore, USP18 interacted with TAK1-TAB1 complex and IKKα/β-NEMO complex, respectively. USP18 cleaved the K63-linked polyubiquitin chains attached to TAK1 in a protease-dependent manner. Moreover, USP18 targeted the IKK complex through the regulatory subunit NEMO of IKK, and specifically inhibited K63-linked ubiquitination of NEMO. Mutation analysis revealed direct binding of USP18 to the UBAN motif of NEMO. Our study has identified a previously unrecognized role for USP18 in the negative regulation of NF-κB activation by inhibiting K63-linked ubiquitination of TAK1 and NEMO through distinct mechanisms.

The nuclear factor κB (NF-κB) transcription factor has been extensively studied, since its discovery in 1986^1^. NF-κB plays a critical role in regulating immediate responses to pathogens, as well as cell proliferation and survival^2^. In unstimulated cells, NF-κB is sequestered in the cytoplasm by the inhibitory proteins of the IκB family^3^. A variety of stimulators, including cytokines such as tumor necrosis factor (TNF-α), interleukin (IL)-1β, and various Toll-like receptor (TLR) ligands, can activate NF-κB signaling through several key adaptor proteins including RIP1, MyD88, and TRIF^4^. These adaptors act on a series of downstream signaling molecules, such as TRAF2, TRAF3, TRAF5, or TRAF6, which can synthesize multiple polyubiquitin chains targeting themselves and other proteins, serving as a scaffold to recruit TAK1 and other kinases. Next, active TAK1 complex initiates MAPK and NF-κB cascades. In turn, the inhibitor of κB kinases (IKK) complex, which is composed of two catalytic subunits IKKα and IKKβ, as well as the essential regulatory subunit NEMO (also known as IKKγ) are recruited to TAK1 complex and undergo phosphorylation^5^,^6^,^7^. Subsequently, active IKK phosphorylates IκBs at serines 32 and 36, leading to the degradation of IκBs by 26S proteasome pathway^8^. Degradation of IκB allows NF-κB nuclear localization and promotes the transcription of its target genes^9^,^10^.

Ubiquitination plays a key role in the activation of NF-κB pathways. Different types of polyubiquitination processes, including Lys-(K) 63, linear (M1), K48, K11, and K27 chains, which are regulated by many different E3 ligases including TRAFs, βTrCP, and other proteins, have been implicated in NF-κB activation^4^,^7^. For example, cellular inhibitor of apoptosis protein (c-IAP1) and the UbcH5 family of proteins promote K11-linked polyubiquitination of RIP1, leading to its degradation^11^. TAK1 can be activated by TRAF6 and TRIM8 through K63-linked ubiquitination^12^,^13^. Furthermore, diverse types of ubiquitination on NEMO, including K63, K27, and M1 polyubiquitinations, are crucial for IKK activation^14^,^15^,^16^. Recently, unanchored polyubiquitin chains alone were also shown to activate TAK1 and IKK complexes^17^.

Deubiquitination is a reverse process of ubiquitination, performed by deubiquitinating enzymes (DUBs). The human genome contains nearly 100 DUBs, with homology within their USP domains, which is used to cleave the polyubiquitin chains^18^. Several DUBs have been reported to function as crucial negative regulators of NF-κB signaling, tightly controlling inflammatory responses. The tumor suppressor, CYLD, inhibits NF-κB activation in a deubiquitinase-dependent manner, by removing K63-linked ubiquitin chains from a variety of signaling proteins, including TRAF2, TRAF6, and RIP1 in T cells and other immune cells^19^. Another deubiquitinase, A20, negatively regulates TLR-induced NF-κB activation, by removing K63-specific polyubiquitin chains from TRAF6. In addition, A20 removes the K63-linked ubiquitin chains on RIP1 and exerts E3 ligase activity by facilitating K48-linked ubiquitination of RIP1, mediating its subsequent proteasomal degradation^20^,^21^. In addition, USP4 inhibits TNF-α-induced activation of NF-κB through USP4 deubiquitination of TAK1^22^. With the exception of CYLD, A20, and USP4, little is known about the proteins responsible for removing different types of polyubiquitin chains from TAK1 and IKK complexes to dampen a robust inflammatory response.

USP18 (also known as UBP43) was originally identified as a type I interferon responsive gene, which is rapidly upregulated by IFN-β treatment through the JAK/STAT kinase pathway^23^. USP18 efficiently cleaves ISG15 conjugates, maintaining cellular homeostasis of ISG15-conjugated proteins^24^. USP18 also negatively regulates type I IFN signaling, independent of its ISG15 isopeptidase activity^25^,^26^. USP18 can specifically bind to the IFNAR2 receptor subunit, to disrupt the interaction between JAK and the IFN receptor, and to inhibit the phosphorylation of receptor-associated JAK1^26^. Recently, USP18 was reported to regulate T helper 17 (Th17) cell differentiation by suppressing the ubiquitination of the TAK1–TAB1 complex^27^. However, the role of USP18 in TLR-induced signaling and inflammation is not clear.

In this study, we identified USP18 as a negative regulator of TLR-induced NF-κB activation through a luciferase assay screening system. USP18 was upregulated by various TLR ligands in THP-1 (a human monocyte cell line) cells and inhibited IκB degradation as well as NF-κB activation to form a negative feedback loop. Knockdown of USP18 markedly enhanced the expression of proinflammatory cytokines in THP-1 cells. Moreover, we found that USP18 targeted TAK1 and IKK complex and specifically inhibited the K63-linked ubiquitination of TAK1 and NEMO through distinct mechanisms. USP18 cleaved the K63-linked polyubiquitin chains of TAK1 in a protease-dependent manner. Conversely, USP18 inhibited NEMO ubiquitination by directly binding to its UBAN motif (ubiquitin binding in ABIN and NEMO) and masking its ubiquitination sites at Lys 325 and 326 from further K63-linked ubiquitination. Our study has identified a previously unrecognized role for USP18 in controlling NF-κB signaling by inhibiting K63-linked polyubiquitination of TAK1 and NEMO, thus negatively regulating the TLR-mediated innate immune response.

## Results

### USP18 negatively regulates TLR-induced NF-κB activation

**Figure 1:**
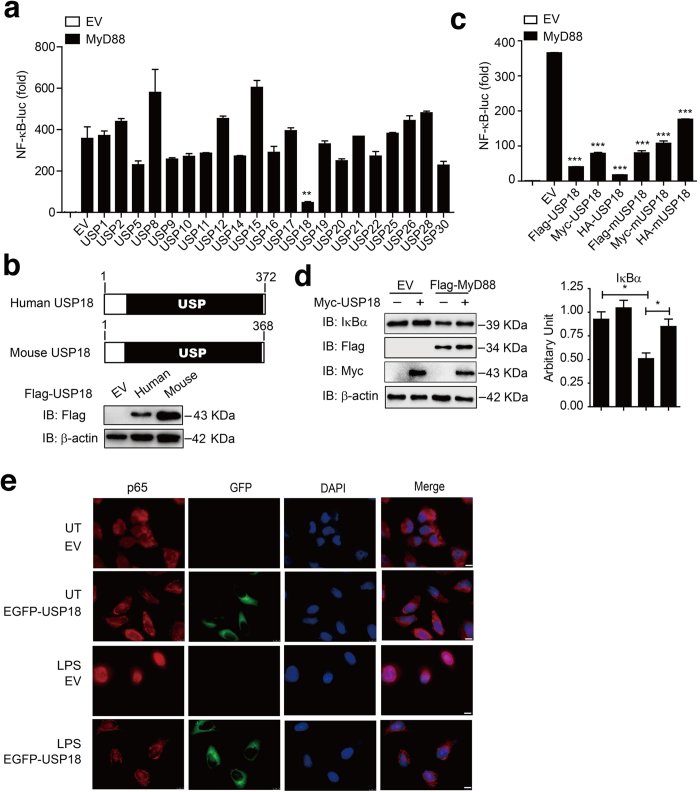
USP18 negatively regulates TLR-induced NF-κB activation. (**a**) HEK293T cells were transfected with plasmids of 21 DUBs along with MyD88 and a reporter plasmid carrying the NF-κB promoter (NF-κB-luc). The cells were analyzed for NF-κB activity by a reporter gene assay. (**b**) Domain organization and immunoblot analysis of human and mouse USP18 proteins. (**c**) 293T cells were transfected with Flag-MyD88 and NF-κB reporter along with different tagged human or mouse USP18 expression plasmids. The cells were analyzed for NF-κB activity by a reporter gene assay. (**d**) Myc-USP18 and Flag-MyD88 were transfected in 293T cells and IκBα turnover was monitored by western blotting using indicated antibodies. (**e**) HeLa cells were transfected with EGFP-USP18 expression plasmid for 48 hrs. Cells were then treated with LPS (100 ng/ml) for 30 min, and then subjected to immunofluorescence analysis using a p65-specific polyclonal antibody. DNA was stained by DAPI (blue). UT, untreated. Scale bar: 10 μm. Data in a,c are presented as the means ± SD of three independent experiments. *P < 0.05, **P < 0.01 and ***P < 0.001, versus cells with the same treatment without USP18 expression (Student’s t-test).

Most TLRs use MyD88 as a pivotal adaptor to activate NF-κB signaling. To identify potential DUBs that can regulate TLR-induced NF-κB signaling, we screened 21 candidate genes encoding DUBs using a MyD88-mediated NF-κB luciferase reporter activation assay in HEK293T (human embryonic kidney 293T) cells. Of these 21 candidate genes, we identified USP18 as a potent negative regulator of MyD88-mediated NF-κB activation (Fig. 1a). Human and mouse USP18 both contain a functional USP domain and share 70% amino acid sequence identity (Fig. 1b). Luciferase assay showed that both human and mouse USP18 markedly inhibited MyD88-mediated NF-κB-luc activation, suggesting a conserved biological function in regulating the NF-κB signaling pathway (Fig. 1c). More importantly, we found that USP18 significantly inhibited the degradation of endogenous IκBα protein in the presence of MyD88 (Fig. 1d). Since the degradation of IκBα releases p65 for nuclear translocation and for the transcription of its target genes^28^, we tested whether USP18 affects the subcellular localization of p65 upon stimulation. Consistent with previous reports^28^, immunofluorescence analysis revealed that activation of the NF-κB signaling pathway by lipopolysaccharide (LPS) treatment of HeLa cells induced the nuclear translocation of p65 from the cytoplasm. Conversely, p65 was predominantly sequestered in the cytoplasm in EGFP-USP18-transfected cells after LPS stimulation (Fig. 1e). Taken together, these results suggest that USP18 inhibits TLR-induced NF-κB activation by blocking the degradation of IκBα as well as by blocking the nuclear accumulation of p65.

### Knockdown of USP18 enhances NF-κB activation as well as the inflammatory response

**Figure 2:**
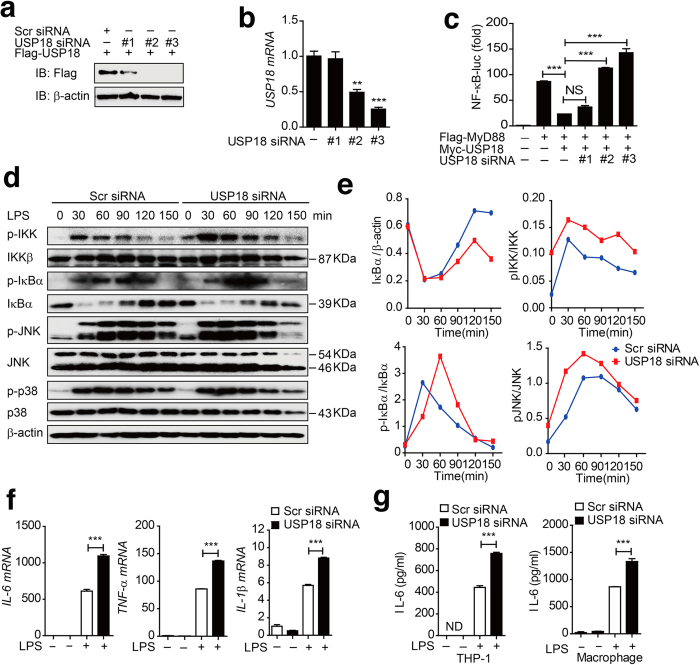
Knockdown of USP18 enhances NF-κB activation as well as the inflammatory response. (**a**) The knockdown efficiency of USP18-specific siRNA. Immunoblot analysis of the knockdown of exogenous USP18 in HEK293T cells expressing Flag-USP18 or endogenous USP18 in THP-1 cells treated with USP18-specific siRNA or scrambled (Scr) siRNA. β-actin serves as a loading control. (**b**) The knockdown efficiency of USP18-specific siRNA at the mRNA level. Real-time PCR analysis of endogenous USP18 in HEK293T cells treated with USP18-specific siRNA or scrambled (Scr) siRNA. (**c**) Luciferase activity in HEK293T cells transfected with USP18-specific siRNA or scrambled (Scr) siRNA, and then transfected with Flag-MyD88 and Myc-USP18 or EV together with an NF-κB luciferase reporter. (**d**) THP-1 cells were transfected with scrambled siRNA or USP18-specific siRNA, and pre-treated with LPS for 12 hrs, followed by the treatment of LPS for the indicated time points. LPS-induced IKK, IKBα and MAPK (JNK and p38) activation were measured by immunoblotting with the indicated antibodies. (**e**) Quantitative comparison of signaling activation between USP18 knockdown and control cells by density scanning of the blots in (**d**). (**f**) THP-1-derived macrophage cells were transfected with Scr siRNA or USP18 siRNA (30 pmol/well) for 48 hrs. The cells were treated with LPS (100 ng/ml) for 2 hrs. The total mRNA was harvested, and IL-1β, IL-6, TNF-α mRNA abundance were analyzed by q-PCR. (**g**) USP18 was knocked down in THP-1 cells and THP-1-derived macrophages. IL-6 and TNF-α production was measured by ELISA after LPS treatment. Data in b, c, f, g are presented as the means ± SD of three independent experiments. *P < 0.05, **P < 0.01, and ***P < 0.001 versus cells transfected with scrambled siRNA (Student’s t-test).

To determine whether specific knockdown of endogenous USP18 would enhance NF-κB activation under physiological conditions, we selected three USP18-specific small interfering RNAs (siRNA) to knock down USP18 expression. Two of the three USP18 siRNAs efficiently inhibited the expression of transfected USP18 and endogenous USP18 in 293T cells and THP-1 cells (Fig. 2a–b). Using the NF-κB luciferase reporter assay, we found that knockdown of USP18 reversed the inhibitory effect of Myc-USP18 on MyD88-mediated NF-κB activation (Fig. 2c). To further confirm the function of USP18 in human macrophages, we knocked down USP18 in THP-1-derived macrophages and stimulated the cells with LPS (Supplementary Fig. S1a). Our results showed that specific knockdown of USP18 resulted in enhanced phosphorylation of IKK and IκBα. In addition, phosphorylation of the MAPK kinase, JNK, but not p38, was also enhanced in cells transfected with USP18-specific siRNA (Fig. 2d–e). Similar results were obtained upon TNF-α treatment of THP-1 cells transfected with USP18 siRNA (Supplementary Fig. S1b). It has been reported that USP18 specifically binds to the IFNAR2 receptor subunit, inhibits the interaction between JAK and the IFN receptor, and attenuates the activity of receptor-associated JAK1^26^. Consistently, we found that knockdown of USP18 in THP-1-derived macrophages also resulted in higher expression levels of several interferon-stimulated genes, including ISG15, IFIT1 (which encodes ISG-54), and IFIT2 (which encodes ISG-56), following treatment with IFN-α (Supplementary Fig. S1c). In addition, we found that LPS treatment resulted in higher expression levels of TNF-α, IL-6, and IL-1β mRNA in THP-1-derived macrophages transfected with USP18-specific siRNA than in those transfected with scrambled siRNA (Fig. 2f). To demonstrate the effects of USP18 on the secretion of proinflammatory cytokines, we knocked down USP18 in THP-1 cells or THP-1-derived macrophages, and then treated the cells with LPS. Knockdown of USP18 resulted in markedly increased secretion levels of IL-6 and TNF-α in both THP-1 moncytes and THP-1and derived macrophages (Fig. 2g). Hence, knockdown of USP18 enhances NF-κB activity, thus increasing NF-κB-dependent proinflammatory cytokine responses in human monocytes and macrophages.

### USP18 inhibits NF-κB signaling at the level of the IKK complex

**Figure 3:**
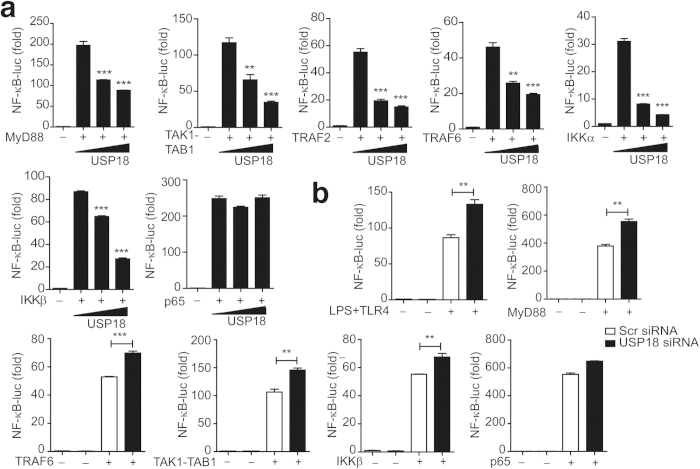
USP18 inhibits NF-κB signaling at the level of the IKK complex. (**a**) USP18 inhibits NF-κB activation induced by NF-κB pathway downstream signaling molecules. 293T cells were transfected with an NF-κB luciferase reporter, together with vector for MyD88, TAK1-TAB1, TRAF2, TRAF6, IKKα, IKKβ, or p65, along with empty vector (no wedge) or increasing amounts (wedge) of expression vector for USP18. (**b**) Knockdown of endogenous USP18 enhanced TNF-α, LPS, MyD88, TRAF6, TAK1, and IKKβ induced NF-κB activation. Luciferase activity in HEK293T cells transfected with USP18-specific or scrambled siRNA and transfected with Flag-MyD88, TRAF6, TAK1+TAB1, IKKβ, p65 or treated with TNF-α or LPS (in 293T-TLR4 cells) together with an NF-κB luciferase reporter. Data in a-b are presented as the means ± SD of three independent experiments. *P < 0.05, **P < 0.01, and ***P < 0.001 versus cells transfected with control vector or scrambled siRNA (Student’s t-test).

To determine the molecular mechanisms by which USP18 inhibits TLR induced NF-κB signaling, we transfected 293T cells with MyD88, TRAF2, TRAF6, TAK1-TAB1, IKKα, IKKβ, or p65 subunit together with increasing amounts of USP18 plus the NF-κB luciferase reporter. We found that the activation of NF-κB by MyD88, TRAF2, TRAF6, TAK1-TAB1, IKKα and IKKβ was markedly inhibited by USP18 (Fig. 3a). In contrast, USP18 did not inhibit p65-mediated NF-κB activation (Fig. 3a), suggesting that USP18 inhibits the NF-κB pathway upstream of p65, most likely targeting the IKK complex. In addition, we found that NF-κB activation through RIG-I(N), MAVS, or TBK1, which do not signal through TAK1, could also be inhibited by USP18 (Supplementary Fig. S2). Consistent with these results, we found that knockdown of USP18 enhanced NF-κB-luc activity induced by TNF-α, LPS, MyD88, TRAF6, TAK1-TAB1, IKKβ, but not p65 (Fig. 3b). These results suggest that USP18 inhibits NF-κB signaling upstream of p65, at the level of the IKK complex.

### USP18 interacts with TAK1-TAB1 complex and the IKK complex

**Figure 4:**
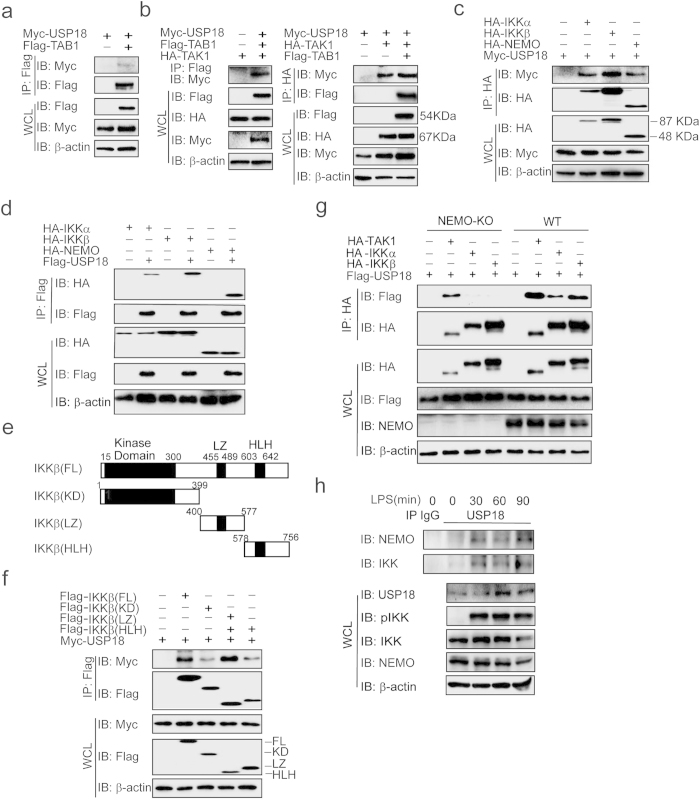
USP18 interacts with TAB1-TAK1 complex and IKK complex. (**a-b**) USP18 interacts with TAB1 and TAK1. Co-immunoprecipitation and immunoblot analysis of 293T cells transfected with various combinations (above lanes) of plasmids for Flag-TAB1, HA-TAK1, and Myc-USP18. (**c**) USP18 interacts with IKKα/β and NEMO. 293T cells were transfected with HA-IKKα, HA-IKKβ, HA-NEMO, and Myc-USP18. HA-tagged IKK protein was immunoprecipitated with anti-HA beads and blotted with anti-Myc. (**d**) 293T cells were transfected with HA-IKKα, HA-IKKβ, HA-NEMO, and Flag-USP18. Flag-tagged USP18 was immunoprecipitated with anti-Flag beads, and blotted with HA. (**e**) The domain structure of IKKβ. Numbers in parentheses indicate amino acid position in construct. KD, kinase domain; LZ, leucine zipper; HLH, helix-loop-helix. (**f**) The interaction between Myc-USP18 and Flag-IKKβ domain. 293T cells were transfected with Myc-USP18 and Flag-IKKβ or various Flag-IKKβ domains. Whole cell extracts were immunoprecipitated with anti-Flag beads, and blotted with anti-Myc. (**g**) NEMO is essential for the interaction between USP18 and IKK. Wild type (WT) 293T cells and NEMO knockout (KO) 293T cells were transfected with Flag-USP18 and HA-TAK1, HA-IKKα or HA-IKKβ. Whole cell extracts were immunoprecipitated with anti-HA beads, and blotted with anti-Flag. (**h**) Immunoassay of extracts of THP-1 derived macrophages treated for various times (above lanes) with LPS, followed by immunoprecipitation with anti-USP18 and immunoblot analysis (antibodies, left margin).

A recent study showed that USP18 targets the TAK1-TAB1 complex for deubiquitination in Th17 cells^27^. We also found that USP18 weakly interacts with TAB1 alone (Fig. 4a), but it has higher affinity for the TAK1-TAB1 complex in the presence of TAK1 (Fig. 4b). More importantly, results presented in Fig. 3 suggest that USP18 may directly interact with the IKK complex to inhibit NF-κB activation. To test this prediction, we transfected 293T cells with USP18 together with IKKα, IKKβ, or NEMO expression plasmids and co-immunoprecipitation and immunoblot analysis revealed that USP18 interacted with IKKα, IKKβ, and NEMO (Fig. 4c–d). To determine the mechanism of the USP18 and IKKβ interaction, we generated deletion mutants encompassing the amino-terminal kinase domain (KD), leucine zipper domain (LZ), and a C-terminal helix-loop-helix (HLH) domain of IKKβ (Fig. 4e), and performed immunoprecipitation to test their ability to interact with USP18. Similar to full-length IKKβ, all mutated domains could interact with USP18 (Fig. 4f), suggesting that IKKβ may not be the direct target of USP18. Next, we examined whether USP18 directly interacts with NEMO. We co-transfected USP18 with TAK1, IKKα, or IKKβ in wild type (WT) 293T and NEMO knockout 293T cells, and found no USP18 interaction with IKKα/β in NEMO knockout 293T cells (Fig. 4g). Therefore, NEMO is essential for the interaction between USP18 and IKKα/β. However, NEMO deficiency did not lead to loss of interaction between USP18 and TAK1 (Fig. 4g), suggesting that USP18 targets TAK1 complex and IKK complex by distinct mechanisms. To further confirm endogenous interaction between USP18 and NEMO, we stimulated THP-1 derived macrophages with LPS for various time points. Little binding between USP18 and NEMO or between USP18 and IKKβ was observed in unstimulated cells. However, these interactions markedly increased after LPS stimulation (Fig. 4h). Taken together, these results suggest that in addition to the TAK1-TAB1 complex, USP18 also interacts with the IKK complex upon LPS treatment in a NEMO-dependent manner.

### USP18 inhibits the K63-linked ubiquitination of TAK1 in a protease-dependent manner

**Figure 5:**
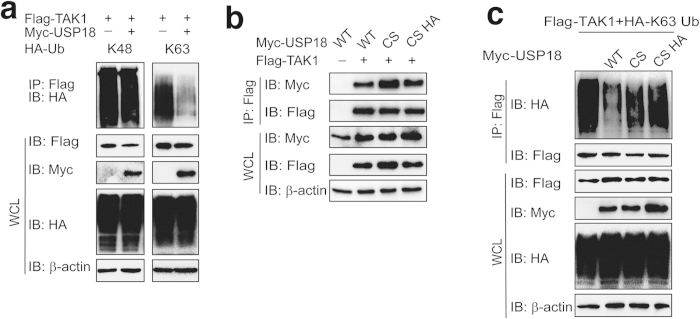
USP18 inhibits the K63-linked ubiquitination of TAK1 in a protease dependent manner. (**a**) USP18 overexpression leads to deubiquitination of TAK1. 293T cells were transfected with Fag-TAK1, Myc-USP18, HA-tagged K48 or K63 ubiquitin mutants. Flag-immunoprecipitation was performed and analyzed using anti-HA antibody by immunoblot analysis. (**b**) USP18 inactive mutants interact with TAK1. 293T cells were transfected with Flag-TAK1 and Myc-USP18 (WT), Myc-USP18 (C64S), or Myc-USP18 (C64S H318A) for 24 hrs. Co-immunoprecipitation and immunoblot analyses were performed with the indicated antibodies. (**c**) USP18 mutation can not cleave the K63-linked poly-ubiquitin chains on TAK1. Co-immunoprecipitation and immunoblot analysis of 293T cells transfected with Flag-TAK1, HA-K63-ubiquitin and Myc-USP18 (WT), Myc-USP18 (C64S) or Myc-USP18 (C64S H318A).

Previous studies showed that USP18 potently abolishes the polyubiquitination of TAK1-TAB1 complex^27^. We further demonstrated that USP18 could remove K63- but not K48-linked polyubiquitin chains from TAK1 (Fig. 5a). To further investigate whether deubiquitination of TAK1 by USP18 is required for USP18 protease activity, we generated protease inactive USP18 mutants by substituting a serine residue for cysteine within the catalytic domain (C64S), and by further substituting the conserved histidine residue with alanine at position 318 (C64S H318A). We found that although USP18 mutants have higher affinity to TAK1 than wild type USP18 (Fig. 5b), neither USP18 (C64S) mutant nor USP18 (C64S H318A) mutant could cleave the K63-linked polyubiquitin chain on TAK1 (Fig. 5c). This suggests that catalytic activity of USP18 is essential for TAK1 deubiquitination.

### USP18 inhibits the conjugated K63-linked ubiquitination of NEMO in a protease-independent manner

**Figure 6:**
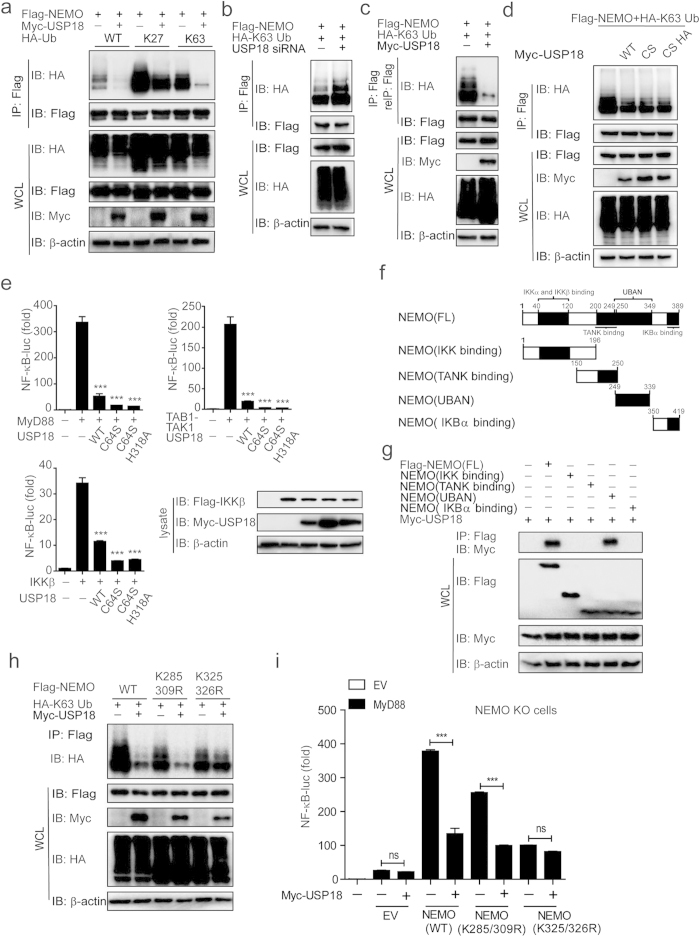
USP18 blocks conjugated K63-linked poly-ubiquitin chains of NEMO at sites of Lys 325 and 326 within the UBAN domain. (**a**) USP18 blocks K63-linked polyubiquitination of NEMO. Lysates of 293T cells transfected with plasmids were immunoprecipitated with anti-Flag and immunoblotted with anti-HA. (**b**) Knockdown of USP18 increased K63-linked ubiquitination of NEMO. Immunoprecipitation and immunoblot analyses were performed after co-transfection of Flag-NEMO, HA-K63 ubiquitin, along with USP18 siRNA or scrambled siRNA in 293T cells. (**c**) Lysates of 293T cells transfected with plasmids were immunoprecipitated anti-Flag, then boiled proteins in presence of 1% SDS, followed by a second immunoprecipitation with anti-Flag. (**d**) USP18 mutations were able to inhibit K63 linked ubiquitination of NEMO. Co-immunoprecipitation and immunoblot analysis of 293T cells transfected with Flag-NEMO, HA-K63 ubiquitin, and Myc-USP18 (WT), Myc-USP18 (C64S), or Myc-USP18 (C64S H318A). (**e**) The inhibition of USP18 in NF-κB is independent of its protease activity. 293T cells were transfected with an NF-κB luciferase reporter, together with vector for MyD88, TAK1+TAB1, IKKβ, along with wild type USP18 or its mutants. The expression of the IKKβ and USP18 mutants were shown by immunoblot. (**f**) The domain structure of NEMO. (**g**) UBAN domain of NEMO is essential for its interaction with USP18. The Flag-tagged different deletion constructs of NEMO were co-transfected with Myc-USP18 in 293T cells and immunoprecipitation and immunoblot were performed. (**h**) Immunoprecipitation and immunoblot analysis of 293T cells transfected with various combinations (above lanes) of plasmid for Myc-tagged USP18 and HA-tagged K63-linked ubiquitin together with plasmid for Flag-tagged NEMO mutations. (**i**) USP18 cannot inhibit NF-κB activation in NEMO knockout cells which transfected with NEMO (K325/326R). NEMO knockout cells were transfected with NEMO (WT), NEMO (K285/309R) mutant or NEMO (K325/326R) mutant and other indicated plasmids. Data in i are presented as the means ± SD of three independent experiments. ***P < 0.001 versus cells transfected with control vector or USP18 (Student’s t-test).

We next examined whether USP18 affects NEMO ubiquitination state via USP18 protease activity. It has been shown that NEMO binds to both K63-linked polyubiquitin chains^29^ and linear polyubiquitin chains via its UBAN motif^16^ for NF-κB activation. As shown in Fig. 6a and Supplementary Fig. S3a-b, overexpression of USP18 markedly inhibited K63-linked ubiquitination of NEMO, but had little or no effect on the ubiquitination of NEMO with the linear (M1) or other linkages (K6, K11, K27, K29, K33, and K48). Consistent with this observation, knockdown of USP18 increased K63-linked ubiquitination of NEMO (Fig. 6b).

Since both conjugated and free polyubiquitin chains have been reported to activate NF-κB signaling, we next investigated which types of polyubiquitin chains on NEMO could be inhibited by USP18. First, we used the two-step immunoprecipitation assay to assess whether USP18 prevents the conjugated ubiquitination of NEMO. Cell lysates were subjected to immunoprecipitation with anti-Flag beads. Next, the immunoprecipitates were denatured, followed by re-immunoprecipitation with anti-Flag beads, to detect only conjugated polyubiquitination modifications of NEMO by immunoblot analysis^17^. We found that USP18 prevented covalently conjugated K63-linked polyubiquitin chains on NEMO (Fig. 6c). To further demonstrate the types of polyubiquitin chains inhibited by USP18, we used IsoT (USP5) to specifically cleave free (unanchored) polyubiquitin chains^30^. We found that USP18 could further inhibit K63-linked ubiquitination of NEMO in the presence of IsoT (Supplementary Fig. S3c), suggesting that USP18 primarily blocked the linkage of conjugated K63-linked polyubiquitin chains on NEMO. Since previous studies showed that NEMO D311N, E315A, and R319Q mutants were unable to bind free ubiquitin chains^31^,^32^, we generated these three mutants and found that USP18 could still inhibit K63-linked ubiquitination of all three mutants (Supplementary Fig. S3d-e). These results indicated that USP18 only inhibits covalently conjugated K63-linked ubiquitin chains attached to NEMO.

We next tested whether USP18 inhibited NEMO ubiquitination through USP18 protease activity. USP18 (C64S) and USP18 (C64S H318A) mutants demonstrated similar binding affinities to NEMO and IKKβ compared with wild type (WT) USP18 (Supplementary Fig. S4a-b). Unlike TAK1, we found that both USP18 (C64S) and USP18 (C64S H318A) mutants maintain their ability to block NEMO ubiquitination similar to wild type USP18 (Fig. 6d), suggesting protease-independent inhibition of NEMO K63-linked ubiquitination. Consistently, we found that both mutants of USP18 have the ability to inhibit MyD88, TAK1-TAB1, and IKKβ induced NF-κB-luc activation (Fig. 6e). These results indicate that USP18 inhibits NEMO ubiquitination as well as NF-κB activity in a protease-independent manner.

### USP18 inhibits NEMO ubiquitination at the Lys -325 and 326 sites by masking the UBAN domain of NEMO

To investigate the precise mechanisms mediating inhibition of NEMO ubiquitination by USP18, we generated NEMO deletion mutants (Fig. 6f), containing a domain responsible for IKK binding, TANK binding, UBAN, or IκBα binding. Co-immunoprecipitation experiments showed that USP18 only interacted with full length (FL) NEMO or its UBAN domain (Fig. 6g). Since the UBAN domain of NEMO contains all five K63-linked ubiquitination sites (Lys 285, Lys 321, Lys 325, Lys 326, and Lys 399)^33^ and two linear ubiquitination sites for NEMO (Lys 285 and Lys 309)^16^, USP18 may prevent NEMO ubiquitination by masking these ubiquitination sites through direct binding. In order to confirm this hypothesis, we substituted Lys 285, 309, 321, 325, 326, and 399 of NEMO with arginine to construct six mutations and found that USP18 can interact with all these mutants (Supplementary Fig. S4c). We next tested whether USP18 affects the K63-linked ubiquitination of these NEMO mutants. We found that all NEMO mutants showed weak polyubiquitination compared to WT NEMO (Supplementary Fig. S4d). In addition, we observed that the inhibition of K63-linked ubiquitination of NEMO by USP18 is almost abrogated using NEMO K325R and K326R mutants, but not other NEMO mutants (Supplementary Fig. S4d). This suggests that USP18 may specifically inhibit the K63-linked ubiquitination of NEMO at the Lys −325 and −326 sites. To further clarify this point, we constructed NEMO double mutants NEMO (K325/326R) and NEMO (K285/309R) (as control) and found USP18 inhibited K63-linked ubiquitination of the NEMO (WT) and NEMO (K285/309R) constructs, but not the NEMO (K325/326R) mutant (Fig. 6h). In addition, we transfected NEMO knockout cells with WT NEMO or these NEMO double mutants. NF-κB activation, as assessed by a luciferase assay, was increased in NEMO KO cells transfected with NEMO (WT), and to a lesser extent using both double mutants. More importantly, USP18 only inhibited NF-κB activation in WT NEMO or NEMO (K285/309R) construct, but had no effect on NEMO (K325/326R) construct (Fig. 6i). It should be noted that the stimulating activity of NF-κB signaling by NEMO (K325/326R) was reduced, compared with WT NEMO (Fig. 6i), probably due to the importance of these two sites (K325/K326) for the K63-linked ubiquitination. These results suggested that the suppression of NF-κB signaling by USP18 is mainly dependent on the inhibition of K63-linked polyubiquitination of NEMO on lysine residues at positions 325 and 326. Collectively, our findings revealed that USP18 inhibits TAK1 and NEMO ubiquitination through different mechanisms.

### USP18 is an inducible gene by TLR-mediated signaling

**Figure 7:**
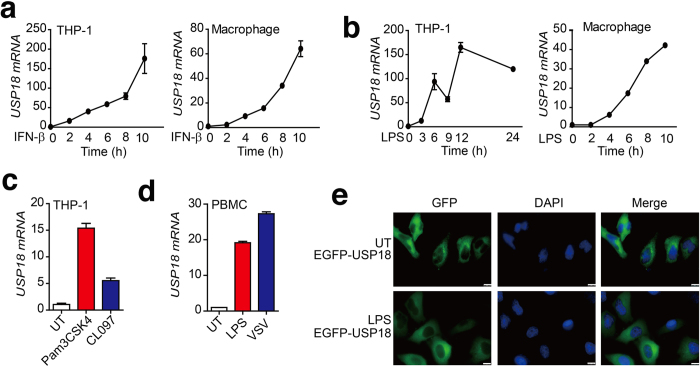
Expression and intracellular localization of human USP18 in response to TLR ligands stimulation. (**a-b**) Relative USP18 mRNA abundance in THP-1 and THP-1-derived macrophage cells were determined by real-time PCR analysis after treatment with IFN-β (10 ng/ml) (**a**) or LPS (200 ng/ml) (**b**) for the indicated time points. (**c**) THP-1 cells stimulated by Pam3CSK4 (200 ng/ml) or CL097 (1 μg/ml). USP18 mRNA abundance was determined by real-time PCR analysis. (**d**) Human peripheral blood mononuclear cells (PBMCs) were treated with LPS or infected with VSV, and USP18-mRNA was determined by real-time PCR analysis. (**e**) Subcellular localization of human USP18 in HeLa cells, with or without LPS treatment. HeLa cells transfected with EGFP-USP18 plasmid were counterstained with DAPI and photographed under a fluorescence microscope. UT, untreated. Scale bar: 10 μm.

Previous studies have shown that the expression of USP18 could be rapidly induced by interferon, viral infection, and genotoxic stress^24^. Consistent with these reports, we observed a strong increase in USP18 mRNA after IFN-β treatment in both THP-1 cells and THP-1-derived macrophages (Fig. 7a). To further validate whether USP18 was regulated by TLR ligand stimulation, we treated THP-1 and THP-1-derived macrophage cells with LPS (a TLR4 ligand). Real-time PCR analysis showed that LPS could rapidly enhance the expression of proinflammatory genes, such as IL-6 and TNF-α (Supplementary Fig. S5). In addition, we found that USP18 mRNA level was significantly increased after LPS stimulation in both THP-1 and THP-1 derived macrophages (Fig. 7b). Other TLR ligands, such as Pam3CSK4 (TLR2 ligand) or CL097 (TLR7/8 ligand), could also induce USP18 expression in THP-1 cells (Fig. 7c). Furthermore, LPS treatment and VSV infection of human peripheral blood mononuclear cells (PBMC) led to increased levels of USP18 mRNA (Fig. 7d). However, LPS treatment did not lead to changes in USP18 cellular localization (Fig. 7e). These results indicate that USP18 expression can be induced by TLR-induced signaling pathways, which, in turn, can suppress TLR-mediated NF-κB activation to form a negative feedback loop.

## Discussion

Activation of NF-κB signaling pathway leads to the production of proinflammatory cytokines, such as IL-6, TNF-α and IL-1β, which, in turn, promote inflammation and induce subsequent adaptive immune responses. Increasing evidence indicates that dysregulated innate immunity may result in many inflammation-associated diseases^34^. Moreover, proinflammatory cytokines produced by innate immune cells in chronic inflammation conditions have been shown to play decisive roles in tumor development^35^,^36^. Therefore, NF-κB should be tightly regulated through multiple negative regulators to avoid persistent inflammatory responses.

Deubiquitinases cleave polyubiquitin chains and play an important role in the termination of NF-κB signaling. CYLD associates with zinc-finger of NEMO, cleaves non-K48-linked poly-ubiquitin chains attached to NEMO, and negatively modulates TRAF-mediated activation of IKK^37^. After stimulation with TNF-α, A20 rapidly forms a complex with NEMO and LUBAC and binds to linear poly-ubiquitin chains via its C-terminal zinc finger 7 to inhibit NF-κB activation^38^. However, it is unknown which DUB is specifically responsible for reversing K63-linked ubiquitination of NEMO. Our results demonstrated that USP18 negatively regulates TLR-induced NF-κB signaling and inflammatory responses by inhibiting K63-linked polyubiquitination of TAK1 and NEMO in a feedback manner. USP18 was reported to be a negative regulator of IFN signaling^26^. Here, we found that ectopic expression of USP18 suppressed nuclear accumulation of p65 as well as NF-κB activation by LPS treatment. Conversely, knockdown of USP18 in THP-1 and THP-1-derived macrophages enhanced the phosphorylation of IKK, the degradation of IKBα and expression of proinflammatory cytokines, such as IL-6, TNF-α and IL-1β in response to LPS treatment These results suggest that USP18 is a novel negative regulator of NF-κB signaling.

Several regulatory proteins of inflammation including, A20, CYLD, and IκB family are direct transcriptional targets of NF-κB, thus forming a negative feedback loop^39^. A previous study showed that USP18 is induced by IFN-β in HO-1 human melanoma cells^23^. We similarly observed the rapid upregulation of USP18 after treatment with IFN-β In addition, we found USP18 is upregulated by TLR2, TLR4, and TLR7/8 ligands in THP-1 cells. Thus, USP18 may link the crosstalk between NF-κB and type I IFN signaling pathways to avoid excessive inflammatory immune responses.

Although a recent study shows USP18 play a regulatory role by targeting TAK1–TAB1 complex for deubiquitination in adaptive immunity^27^. Our results demonstrate the role of USP18 in innate immune cells. USP18 directly cleaves the K63-linked polyubiquitin chains, but not K48-linked polyubiquitin chains from TAK1 in a protease dependent manner since the USP18 catalytically inactive mutant cannot deubiquitinate TAK1. A previous study showed that the UBAN region of NEMO has higher affinity for linear ubiquitin chains than for K63-linked ubiquitin chains^40^. Recently, it was also demonstrated that most of the Met1-linked ubiquitin oligomers formed in response to IL-1β are covalently attached to K63-linked ubiquitin oligomers, indicating that K63-linked ubiquitin oligomers are a prerequisite for LUBAC-catalyzed formation of K63- and Met1- linked polyubiquitin hybrids in IL-1β stimulated cells^41^. Our results demonstrated that USP18 inhibits covalently conjugated K63-linked ubiquitination of NEMO but had little or no effects on the linear (M1) ubiquitination of NEMO. More importantly, co-immunoprecipitation and immunoblot analyses showed that USP18 directly interacts with the UBAN domain of NEMO and masks the ubiquitylation sites at Lys 325 and 326 of NEMO to inhibit the K63-linked polyubiquitination of NEMO. This result suggests that USP18 competes with polyubiquitin chains for NEMO binding, consequently inhibiting its activation and downstream intracellular signaling. The catalytically inactive mutations of USP18 (C64S and H318A) cannot abolish this inhibitory function on NF-κB activation, indicating that USP18 function is dependent on competitive association and is independent of protease activity. Recently, Rhbdd3 has been reported to directly bind to K27-linked polyubiquitin chains on Lys302 of NEMO via its UBA domain in endosomes, and further recruit the A20 to inhibit K63-linked polyubiquitination of NEMO^15^. Our study provides evidence of an alternative mechanism by which K63-linked ubiquitination of NEMO is negatively regulated.

**Figure 8:**
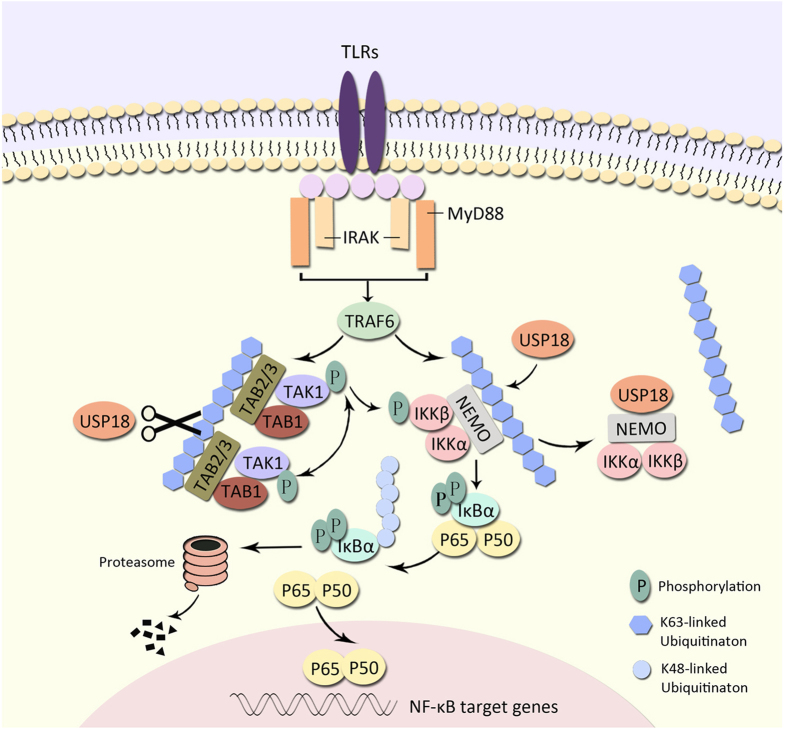
Working model of the negative regulation of the NF-κB pathway by USP18. USP18 interacts with and inhibits the ubiquitination of TAK1 and NEMO in a protease-dependent or protease-independent manner, respectively (Details are as described under “Discussion”).

Based on our experimental data, we propose a working model to explain how USP18 negatively regulates NF-κB signaling by targeting TAK1 and NEMO. USP18 inhibits the K63-linked ubiquitination of TAK1 and NEMO through distinct mechanisms (Fig. 8). After TLR ligand stimulation, TAK1 and NEMO undergo K63-linked ubiquitination by TRAF6, while upregulated USP18 targets TAK1 and NEMO. On one hand, USP18 interacts with TAK1 and cleaves the K63-linked polyubiquitin chains on TAK1, which is dependent on its protease activity. On the other hand, USP18 inhibits NEMO ubiquitination by directly binding to its UBAN domain. Although USP18 mutants can still bind to ubiquitinated TAK1, it fails to cleave the polyubiquitin chains, resulting in a stable USP18-ubiquitinated-TAK1 complex. Thus, USP18 mutants maintain inhibition of NEMO ubiquitination by masking its ubiquitination sites.

It is obvious that NF-κB is controlled by a large amount of positive and negative regulators. Therefore, NF-κB regulation cannot be explained by a single isolated mechanism. Our findings provide additional insight into the molecular mechanisms by which USP18 negatively regulates TLR-induced NF-κB signaling, and thus, plays an important role in the regulation of inflammation.

## Methods

### Cell culture and transfection

HEK293T (human embryonic kidney 293T), HeLa, PBMCs, and THP-1 cells were maintained in DMEM (Hyclone) or RPMI-1640 medium (Gibco) supplemented with 10% fetal bovine serum (Gibco) and 1% L-glutamine (Gibco) at 37 °C in 5% CO2. THP-1 cells were differentiated into macrophages by PMA (100 nM) (InvivoGen) treatment for 12 hrs. Expression plasmids were transfected with Lipofectamine 2000 reagent (Invitrogen) according to the manufacturer’s instructions.

### Antibodies and reagents

Horseradish peroxidase (HRP)-anti-Flag (M2) (A8592) and anti-β-actin (A1978) were purchased from Sigma; HRP-anti-hemagglutinin (12013819001), anti-c-Myc-HRP (11814150001) were purchased from Roche Applied Science; anti-phospho-IKKα/β (No.2697S), anti-phospho-JNK (No.9251), anti-JNK (No.9252), anti-phospho-ERK (No.9101), anti-ERK (No.9102), anti-phospho-p38 (No.9211), anti-p38 (No.9212), anti-p65 (No.6956), anti-IκBα (No.4814) and anti-phospho-IkBα (Ser32/36) (No. 9246) were from Cell Signaling Technology; anti-NEMO (sc-8300), anti-USP18(sc-98431) were purchased from Santa Cruz Biotechnology; anti-IKKβ were purchased from Millipore; CF568 Goat anti-mouse IgG (H+L) (20101-1) were purchased from Biotium; anti-linear polyubiquitin-specific monoclonal antibody (AB130) were purchased from LifeSensors.

Recombinant human TNF-α was purchased from PeproTech; Lipopolysaccharides (LPS) (L4391-1 MG) were purchased from Sigma. Pam3CSK4 and CL097 were purchased from InvivoGen.

### Luciferase reporter assays

HEK293T (5 × 10^4^) cells were plated in 96-well plates and transfected with plasmids encoding an NF-κB (firefly luciferase plasmid; 5 ng), and pRL-TK (renilla luciferase plasmid; 2 ng) together with 10–25 ng plasmid encoding Flag-MyD88, Flag-TRAF6, Flag-TAK1+HA-TAB1, Flag-IKKβ, Flag-IKKα, Flag-p65, Flag-RIG-I(N), Flag-MAVS, Flag-TBK1, and increasing concentrations (0, 50, or 100 ng) of plasmid expressing USP18. Cells were harvested at 24–36 h after transfection in passive lysis buffer (Promega). Cell lysates were measured using the dual luciferase assay kit according to the manufacturer’s protocol (Promega). The enzyme activity was normalized for the efficiency of transfection on the basis of Renilla luciferase activity levels and results are shown as fold induction relative to the basal level measured in cells transfected with empty vector or scrambled siRNA. Values are mean ± SD from three independent transfections performed in parallel.

### Immunoprecipitation and immunoblot analysis

Cells were extracted in ice-cold low-salt lysis buffer (50 mM Hepes pH 7.5, 150 mM NaCl, 1 mM EDTA, 1.5 mM MgCl_2_, 10% glycerol, 1% Triton X-100) supplemented with 5 mg/ml protease inhibitor cocktail (Roche). Protein concentration was measured with BCA Protein Assay Kit (Pierce, Rockford, IL), and samples of 20–35 μg total proteins were subjected to SDS-PAGE. For immunoprecipitation (IP) experiments, whole-cell extracts were prepared after transfection or stimulation with appropriate ligands, followed by incubation overnight with the anti-Flag or anti-hemagglutinin agarose gels (Sigma). Beads were washed three times with low-salt lysis buffer. Immunoprecipitates were resuspended with 3 × SDS Loading Buffer (FD Biotechnology) and boiled for 5 minutes. The released proteins were electrophoresed on 8–12% SDS-polyacrylamide gel and transferred onto PVDF membranes, with subsequent blocking using 5% skim milk. Membranes were incubated with specific antibodies, and detected using enhanced chemiluminescence (Millipore).

### Immunofluorescence assay

Cells grown on dishes were fixed with 4% paraformaldehyde for 15 min, and then permeabilized in methyl alcohol for 10 min at −20 °C. After washing with PBS, cells were blocked in 5% fetal goat serum for 1 h, and then incubated with primary antibodies diluted in 10% bull serum albumin overnight. The cells were washed, and followed by a fluorescently labeled secondary antibody (goat anti-mouse IgG conjugated with CF568). Nuclear DNA was stained using 5 μg/ml 4′,6-diamidino-2-phenylindole (DAPI), a fluorescent DNA-intercalating dye. A radiance microscope system (Leica, Germany) was used to visualize the distribution of p65 or EGFP-USP18.

### Real-Time PCR analysis

Total cellular RNA was isolated by TRIzol Reagent (Invitrogen), and first-strand cDNA was generated from total RNA using oligo-dT primers and reverse transcriptase (TAKARA). Real-time PCR was performed with the SYBR Green qPCR Mix (GenStar) and specific primers using the Primer5 analyzer (Applied Biosystems). Data were normalized to GAPDH gene, and the relative abundance of transcripts was calculated by the Ct models. The following primers were used for real-time PCR:

hUSP18 forward primer, 5′ CCTGAGGCAAATCTGTCAGTC 3′

hUSP18 reverse primer, 5′CGAACACCTGAATCAAGGAGTTA 3′

hGAPDH forward primer, 5′ ACAACTTTGGTATCGTGGAAGG 3′

hGAPDH reverse primer, 5′ GCCATCACGCCACAGTTTC 3′

hIL-1β forward primer, 5′ATGATGGCTTATTACAGTGGCAA 3′

hIL-1β reverse primer, 5′ GTCGGAGATTCGTAGCTGGA 3′

hIL-6 forward primer, 5′ AGAGGCACTGGCAGAAAACAAC 3′

hIL-6 reverse primer, 5 AGGCAAGTCTCCTCATTGAATCC 3′

hTNF-α forward primer, 5′ CCAGACCAAGGTCAACCTCC 3′

hTNF-α reverse primer, 5′ CAGACTCGGCAAAGTCGAGA 3′

hISG15 forward primer, 5′ TCCTGGTGAGGAATAACAAGGG 3′

hISG15 reverse primer, 5′ GTCAGCCAGAACAGGTCGTC 3′

hISG54 forward primer, 5′ GGAGGGAGAAAACTCCTTGGA 3′

hISG54 reverse primer, 5′ GGCCAGTAGGTTGCACATTGT 3′

hISG56 forward primer, 5′ TCAGGTCAAGGATAGTCTGGAG 3′

hISG56 reverse primer, 5′ AGGTTGTGTATTCCCACACTGTA 3′

### Knockdown of USP18 by RNA interference

LipoRNAiMAX (Invitrogen) was used according to the manufacturer’s protocols for transfection of siRNAs into THP-1 cells or THP-1-derived macrophages. The sequences of USP18 siRNAs are as follows:

Human USP18-specific siRNA

#1 SenseSeq: CUGCAUAUCUUCUGGUUUATT

 AntiSeq: UAAACCAGAAGAUAUGCAGTT

#2 SenseSeq: ACAUGAAGAUGGAGUGCUATT

 AntiSeq: UAGCACUCCAUCUUCAUGUTT

#3 SenseSeq: GGAAUUCACAGACGAGAAATT

 AntiSeq: UUUCUCGUCUGUGAAUUCCTT

### Enzyme-linked immunosorbent assay (ELISA)

The concentration of human IL-6 and TNF-α protein in cell culture supernatants was estimated with ELISA kits (BD Biosciences 555220 and 555212) according to the manufacturer’s recommendations.

### Statistical analysis

The results of all quantitative experiments are reported as mean ± SEM of three independent experiments, and Student’s t-test was used for all statistical analyses with the GraphPad Prism 5.0 software. Differences between groups were considered significant when *P* value was less than 0.05.

## Additional Information

**How to cite this article**: Yang, Z. *et al.* USP18 negatively regulates NF-κB signaling by targeting TAK1 and NEMO for deubiquitination through distinct mechanisms. *Sci. Rep.* **5**, 12738; doi: 10.1038/srep12738 (2015).

## Supplementary Material

Supplementary Information

## References

[b1] Sen R. & Baltimore D. Multiple nuclear factors interact with the immunoglobulin enhancer sequences. Cell 46, 705–716 (1986). 3091258 10.1016/0092-8674(86)90346-6

[b2] Ghosh S. & Hayden M. S. New regulators of NF-kappaB in inflammation. Nat Rev Immunol 8, 837–848 (2008). 18927578 10.1038/nri2423

[b3] Scheidereit C. IkappaB kinase complexes: gateways to NF-kappaB activation and transcription. Oncogene 25, 6685–6705 (2006). 17072322 10.1038/sj.onc.1209934

[b4] Iwai K. Diverse ubiquitin signaling in NF-kappaB activation. Trends Cell Biol 22, 355–364 (2012). 22543051 10.1016/j.tcb.2012.04.001

[b5] Chen Z. J., Parent L. & Maniatis T. Site-specific phosphorylation of IkappaBalpha by a novel ubiquitination-dependent protein kinase activity. Cell 84, 853–862 (1996). 8601309 10.1016/s0092-8674(00)81064-8

[b6] Chen Z. J. Ubiquitin signalling in the NF-kappaB pathway. Nat Cell Biol 7, 758–765 (2005). 16056267 10.1038/ncb0805-758 PMC1551980

[b7] Zinngrebe J., Montinaro A., Peltzer N. & Walczak H. Ubiquitin in the immune system. Embo Rep 15, 28–45 (2014). 24375678 10.1002/embr.201338025 PMC4303447

[b8] Maniatis T. A ubiquitin ligase complex essential for the NF-kappaB, Wnt/Wingless, and Hedgehog signaling pathways. Genes Dev 13, 505–510 (1999). 10072378 10.1101/gad.13.5.505

[b9] Baldwin A. J. The NF-kappa B and I kappa B proteins: new discoveries and insights. Annu Rev Immunol 14, 649–683 (1996). 8717528 10.1146/annurev.immunol.14.1.649

[b10] Vallabhapurapu S. & Karin M. Regulation and function of NF-kappaB transcription factors in the immune system. Annu Rev Immunol 27, 693–733 (2009). 19302050 10.1146/annurev.immunol.021908.132641

[b11] Dynek J. N. *et al.* c-IAP1 and UbcH5 promote K11-linked polyubiquitination of RIP1 in TNF signalling. Embo J 29, 4198–4209 (2010). 21113135 10.1038/emboj.2010.300 PMC3018797

[b12] Wang C. *et al.* TAK1 is a ubiquitin-dependent kinase of MKK and IKK. Nature 412, 346–351 (2001). 11460167 10.1038/35085597

[b13] Li Q. *et al.* Tripartite motif 8 (TRIM8) modulates TNFalpha- and IL-1beta-triggered NF-kappaB activation by targeting TAK1 for K63-linked polyubiquitination. Proc Natl Acad Sci USA 108, 19341–19346 (2011). 22084099 10.1073/pnas.1110946108 PMC3228454

[b14] Arimoto K. *et al.* Polyubiquitin conjugation to NEMO by triparite motif protein 23 (TRIM23) is critical in antiviral defense. Proc Natl Acad Sci USA 107, 15856–15861 (2010). 20724660 10.1073/pnas.1004621107 PMC2936632

[b15] Liu J. *et al.* Rhbdd3 controls autoimmunity by suppressing the production of IL-6 by dendritic cells via K27-linked ubiquitination of the regulator NEMO. Nat Immunol 15, 612–622 (2014). 24859449 10.1038/ni.2898

[b16] Tokunaga F. *et al.* Involvement of linear polyubiquitylation of NEMO in NF-kappaB activation. Nat Cell Biol 11, 123–132 (2009). 19136968 10.1038/ncb1821

[b17] Xia Z. P. *et al.* Direct activation of protein kinases by unanchored polyubiquitin chains. Nature 461, 114–119 (2009). 19675569 10.1038/nature08247 PMC2747300

[b18] Chen J. & Chen Z. J. Regulation of NF-kappaB by ubiquitination. Curr Opin Immunol 25, 4–12 (2013). 23312890 10.1016/j.coi.2012.12.005 PMC3594545

[b19] Harhaj E. W. & Dixit V. M. Deubiquitinases in the regulation of NF-kappaB signaling. Cell Res 21, 22–39 (2011). 21119682 10.1038/cr.2010.166 PMC3075605

[b20] Harhaj E. W. & Dixit V. M. Regulation of NF-kappaB by deubiquitinases. Immunol Rev 246, 107–124 (2012). 22435550 10.1111/j.1600-065X.2012.01100.x PMC3540820

[b21] Wertz I. E. *et al.* De-ubiquitination and ubiquitin ligase domains of A20 downregulate NF-kappaB signalling. Nature 430, 694–699 (2004). 15258597 10.1038/nature02794

[b22] Fan Y. H. *et al.* USP4 targets TAK1 to downregulate TNFalpha-induced NF-kappaB activation. Cell Death Differ 18, 1547–1560 (2011). 21331078 10.1038/cdd.2011.11 PMC3136563

[b23] Kang D., Jiang H., Wu Q., Pestka S. & Fisher P. B. Cloning and characterization of human ubiquitin-processing protease-43 from terminally differentiated human melanoma cells using a rapid subtraction hybridization protocol RaSH. Gene 267, 233–242 (2001). 11313150 10.1016/s0378-1119(01)00384-5

[b24] Malakhov M. P., Malakhova O. A., Kim K. I., Ritchie K. J. & Zhang D. E. UBP43 (USP18) specifically removes ISG15 from conjugated proteins. J Biol Chem 277, 9976–9981 (2002). 11788588 10.1074/jbc.M109078200

[b25] Kim K. I. *et al.* Ube1L and protein ISGylation are not essential for alpha/beta interferon signaling. Mol Cell Biol 26, 472–479 (2006). 16382139 10.1128/MCB.26.2.472-479.2006 PMC1346917

[b26] Malakhova O. A. *et al.* UBP43 is a novel regulator of interferon signaling independent of its ISG15 isopeptidase activity. Embo J 25, 2358–2367 (2006). 16710296 10.1038/sj.emboj.7601149 PMC1478183

[b27] Liu X. *et al.* USP18 inhibits NF-kappaB and NFAT activation during Th17 differentiation by deubiquitinating the TAK1-TAB1 complex. J Exp Med 210, 1575–1590 (2013). 23825189 10.1084/jem.20122327 PMC3727316

[b28] Ghosh S., May M. J. & Kopp E. B. NF-kappa B and Rel proteins: evolutionarily conserved mediators of immune responses. Annu Rev Immunol 16, 225–260 (1998). 9597130 10.1146/annurev.immunol.16.1.225

[b29] Wu C. J., Conze D. B., Li T., Srinivasula S. M. & Ashwell J. D. Sensing of Lys 63-linked polyubiquitination by NEMO is a key event in NF-kappaB activation [corrected]. Nat Cell Biol 8, 398–406 (2006). 16547522 10.1038/ncb1384

[b30] Reyes-Turcu F. E. *et al.* The ubiquitin binding domain ZnF UBP recognizes the C-terminal diglycine motif of unanchored ubiquitin. Cell 124, 1197–1208 (2006). 16564012 10.1016/j.cell.2006.02.038

[b31] Windheim M., Stafford M., Peggie M. & Cohen P. Interleukin-1 (IL-1) induces the Lys63-linked polyubiquitination of IL-1 receptor-associated kinase 1 to facilitate NEMO binding and the activation of IkappaBalpha kinase. Mol Cell Biol 28, 1783–1791 (2008). 18180283 10.1128/MCB.02380-06 PMC2258775

[b32] Rahighi S. *et al.* Specific recognition of linear ubiquitin chains by NEMO is important for NF-kappaB activation. Cell 136, 1098–1109 (2009). 19303852 10.1016/j.cell.2009.03.007

[b33] Sebban-Benin H. *et al.* Identification of TRAF6-dependent NEMO polyubiquitination sites through analysis of a new NEMO mutation causing incontinentia pigmenti. Hum Mol Genet 16, 2805–2815 (2007). 17728323 10.1093/hmg/ddm237

[b34] Takeuchi O. & Akira S. Pattern recognition receptors and inflammation. Cell 140, 805–820 (2010). 20303872 10.1016/j.cell.2010.01.022

[b35] Karin M. Nuclear factor-kappaB in cancer development and progression. Nature 441, 431–436 (2006). 16724054 10.1038/nature04870

[b36] Grivennikov S. I., Greten F. R. & Karin M. Immunity, inflammation, and cancer. Cell 140, 883–899 (2010). 20303878 10.1016/j.cell.2010.01.025 PMC2866629

[b37] Trompouki E. *et al.* CYLD is a deubiquitinating enzyme that negatively regulates NF-kappaB activation by TNFR family members. Nature 424, 793–796 (2003). 12917689 10.1038/nature01803

[b38] Verhelst K. *et al.* A20 inhibits LUBAC-mediated NF-kappaB activation by binding linear polyubiquitin chains via its zinc finger 7. Embo J 31, 3845–3855 (2012). 23032186 10.1038/emboj.2012.240 PMC3463847

[b39] Komuro A., Bamming D. & Horvath C. M. Negative regulation of cytoplasmic RNA-mediated antiviral signaling. Cytokine 43, 350–358 (2008). 18703349 10.1016/j.cyto.2008.07.011 PMC2575845

[b40] Lo Y. C. *et al.* Structural basis for recognition of diubiquitins by NEMO. Mol Cell 33, 602–615 (2009). 19185524 10.1016/j.molcel.2009.01.012 PMC2749619

[b41] Emmerich C. H. *et al.* Activation of the canonical IKK complex by K63/M1-linked hybrid ubiquitin chains. Proc Natl Acad Sci USA 110, 15247–15252 (2013). 23986494 10.1073/pnas.1314715110 PMC3780889

