# Supplementary material for: USP18 negatively regulates NF-κB signaling by targeting TAK1 and NEMO for deubiquitination through distinct mechanisms
Source: Sci Rep. 2015 Aug 4;5:12738. doi: 10.1038/srep12738 (PMC4523862; doi:10.1038/srep12738)
Supplement: Supplementary Information [file srep12738-s1.pdf]

Supplementary material

USP18 negatively regulates NF-κB signaling by targeting TAK1 and NEMO for deubiquitination through distinct mechanisms

Zhifen Yang[1, 2+], Huifang Xian[1+], Jiajia Hu[1], Shuo Tian[1], Yunfei Qin[1], Rong-Fu Wang[4*] and Jun Cui[1,3*]

[1] Key Laboratory of Gene Engineering of the Ministry of Education, State Key Laboratory of Biocontrol, School of Life Sciences, [2] Zhongshan School of Medicine, [3] Collaborative Innovation Center of Cancer Medicine, Sun Yat-sen University, Guangzhou, P. R. China. [4]Houston Methodist Research Institute, Houston, Texas 77030, USA

*Correspondence should be addressed to J.C. (cuij5@mail.sysu.edu.cn) and R.-F.W. (rwang3@tmhs.org).

[+]These authors contributed equally to this work.

Figure S1

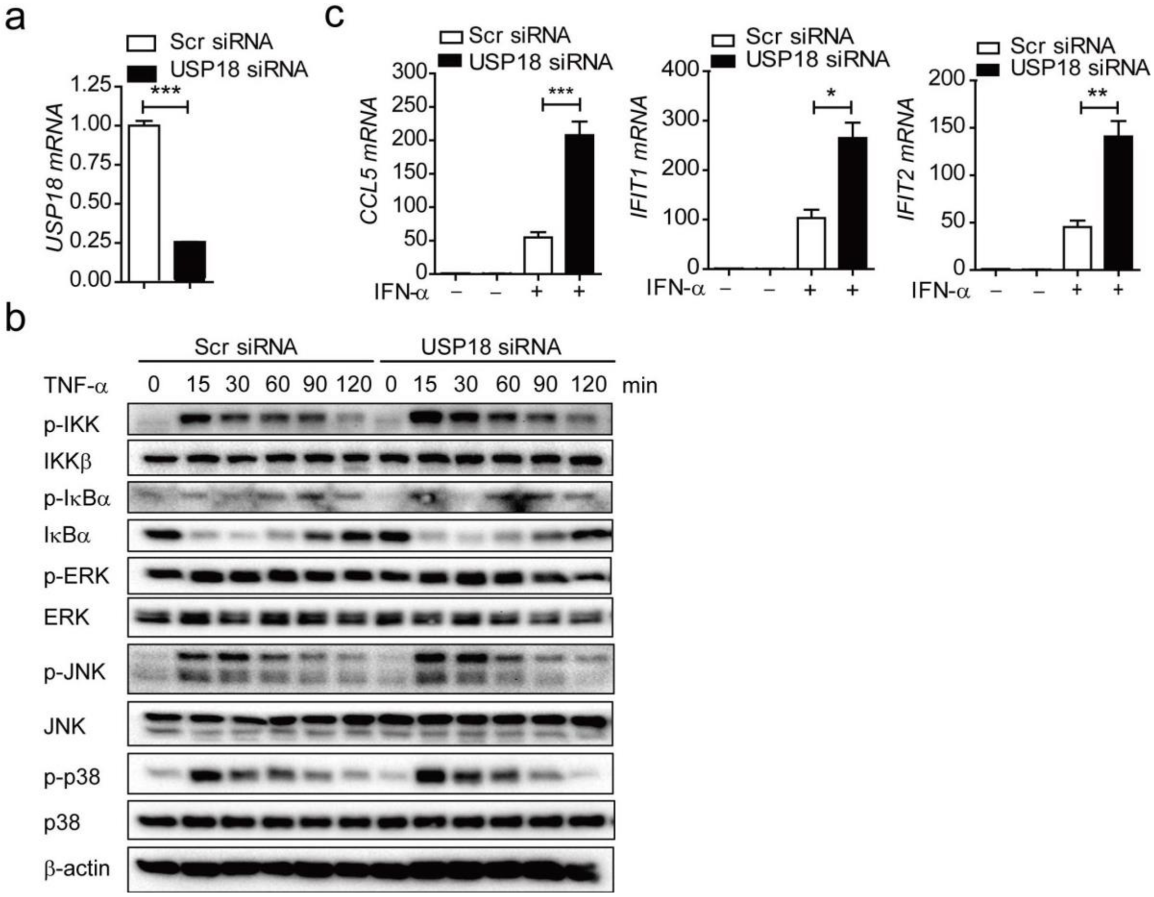

Figure S1 | Knockdown of USP18 enhances activation of NF-κB signaling and expression of interferon-stimulated genes. (a) Knockdown efficiency of USP18-specific siRNA in THP-1 derived macrophages. (b) THP-1 cells transfected with scramble (Scr) siRNA or USP18-specific siRNA, and then treated with TNF-α for the indicated time points. TNFα-induced IKK, IKBα, and MAPK (p38, JNK, and ERK) activation measured by immunoblot analysis with indicated antibodies. (c) USP18-mediated negative regulation of IFN-α induced gene expression. THP-1 derived macrophage cells transfected with Scr siRNA or USP18 siRNA 30 pmol/well for 48 hrs, followed by treatment with IFN-α (10ng/ml) for 6 hrs. Total mRNA harvested after treatment and ISG15, IFIT1, IFIT2 mRNA abundance analyzed by q-PCR. Data in a, c are presented as the means ± SD of three independent experiments. *P < 0.05, **P < 0.01, and ***P < 0.001 versus cells transfected with scrambled siRNA (Student's t-test).

Figure S2

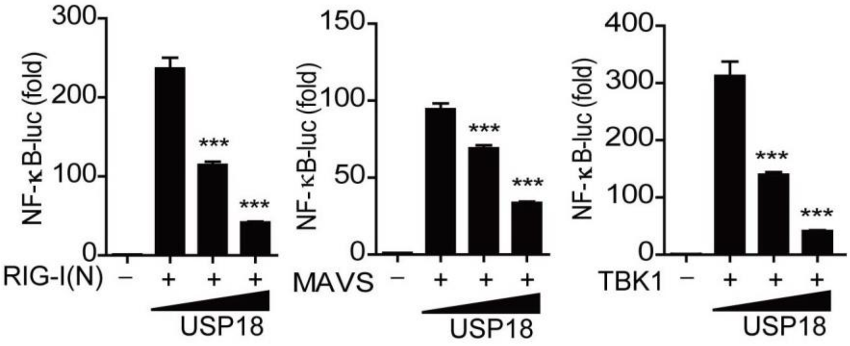

Figure S2 | USP18 inhibits RIG-I (N)-, MAVS-, and TBK1- induced NF-κB activation. Luciferase activity in HEK293T cells transfected with plasmid encoding a luciferase reporter for NF-κB (NF-κB-luc) together with empty vector or plasmids as indicated.

Figure S3

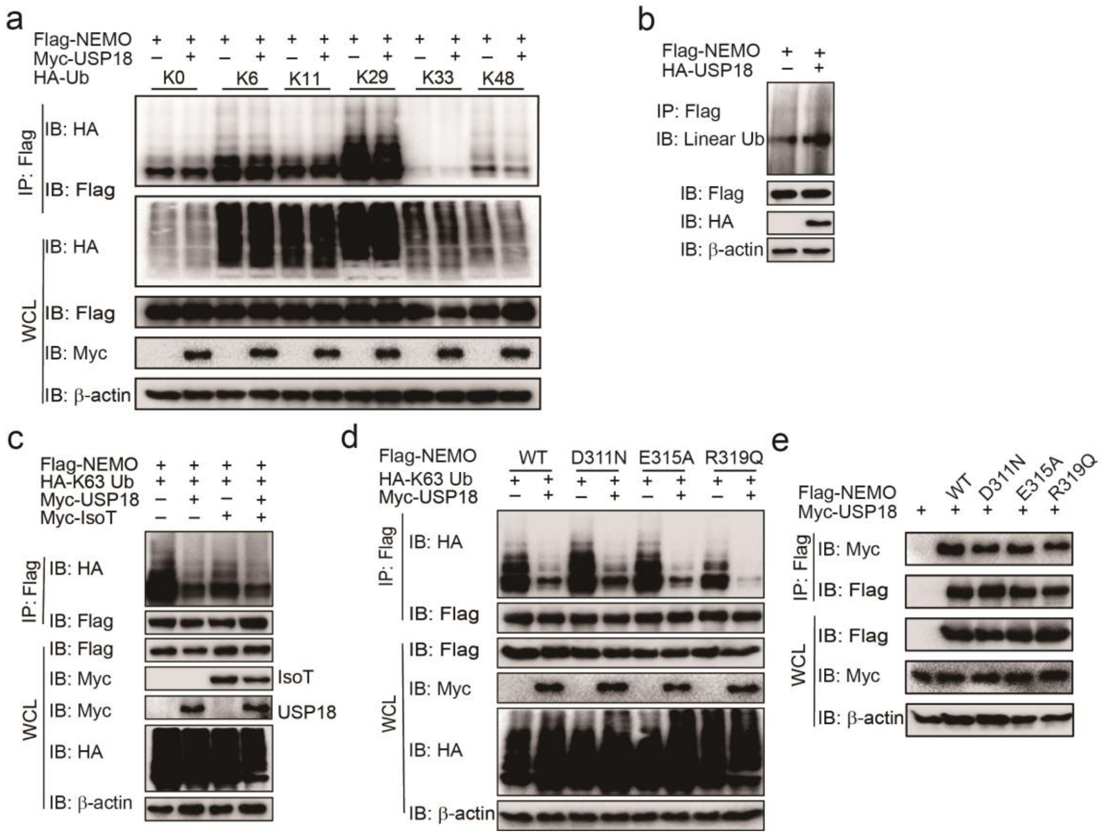

Figure S3 | USP18 blocks covalent conjugated K63-linked polyubiquitin chains of NEMO. (a) Lysates of 293T cells transfected with Flag-NEMO, HA-Ub and Myc-USP18 or EV immunoprecipitated with anti-Flag and immunoblotted with anti-HA. (b) USP18 does not block linear polyubiquitination of NEMO. Lysates of 293T cells transfected with plasmids were immunoprecipitated with anti-Flag and immunoblotted with anti-Linear Ub. (c) Lysates of 293T cells transfected with the indicated plasmids, immunoprecipitated with anti-Flag, and immunoblotted with anti-HA. (d) 293T cells transfected with HA-K63, Flag-NEMO mutants, and Myc-USP18 or EV, then immunoprecipitated with anti-Flag and immunoblotted with anti-HA. (e) Cell lysates from Flag-NEMO mutant- and Myc-USP18-transfected 293T cells immunoprecipitated with anti-Flag and immunoblotted with anti-Myc.

FigureS4

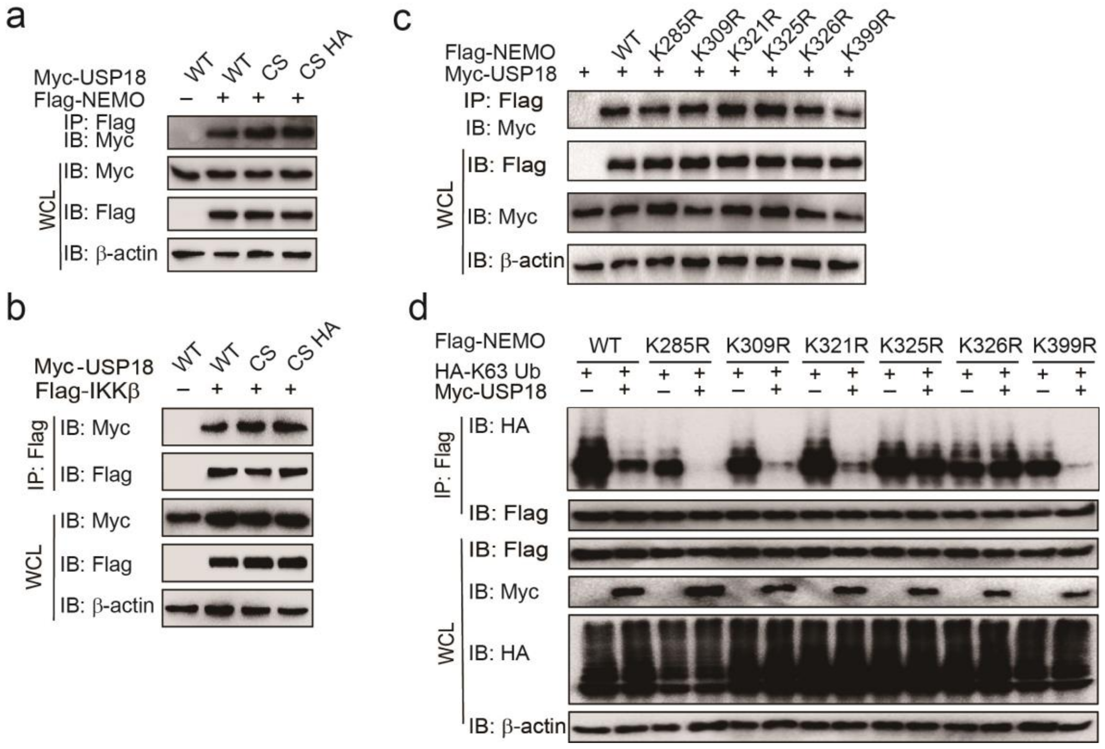

Figure S4 | USP18 blocks K63-linked polyubiquitination of NEMO at Lys 325 and 326 residues. (a-b) Co-immunoprecipitation and immunoblot analyses of 293T cells transfected with Flag-NEMO (a) or IKKβ (b) and Myc-USP18 (WT), Myc-USP18 (C64S), or Myc-USP18 (C64S H318A). (c) 293T cells transfected with Flag-NEMO mutants and Myc-USP18 or EV, immunoprecipitated with anti-Flag, and immunoblotted with anti-Myc. (d) Cell lysates collected from 293T cells transfected with Flag-NEMO mutants and HA-K63 Ub, empty vector (-), or Myc-USP18 were immunoprecipitated with anti-Flag and immunoblotted with anti-HA.

Figure S5

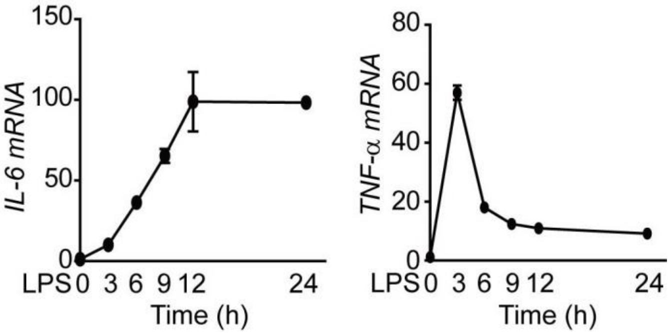

Figure S5 | The expression of proinflammatory cytokines in response to LPS stimulation. Relative IL-6 and TNF-α mRNA abundance in THP-1 cells stimulated with LPS for the indicated time points.
